# To Invalids

**Published:** 1875-11

**Authors:** 


### TO INVALIDS.

Dr. E. H. Gibbs, of this journal, takes pleasure in announcing that he has complied with the requests so frequently made, and has provided a comfortable home within a short distance of his office, where patients will receive careful attention, under his own supervision. The terms for board and medical attendance will be reasonable. Dr. Gibbs is a thoroughly educated and successful physician, and has the advantage of many years' hospital practice in this country and and in Europe, much of the time as physician in charge. He gives his attention to the treatment of chronic affections, such as "Spinal Irritation," "Rheumatism," and nervous diseases. These he treats by a system exclusively his own, which he finds entirely successful in a great majority of cases.

Invalids out of the city can make arrangements for consultation at their own homes.

Address E. H. Gibbs, M.D.,
137 Eighth Street, near Broadway,
New York.

